# Soluble IL-2 Receptor in Dermatomyositis: Its Associations with Skin Ulcers and Disease Activity

**DOI:** 10.1155/2020/6243019

**Published:** 2020-07-28

**Authors:** Linrong He, Xiaoming Shu, Xia Liu, Yongpeng Ge, Sizhao Li, Xin Lu, Guochun Wang

**Affiliations:** Department of Rheumatology, Beijing Key Lab for Immune-Mediated Inflammatory Diseases, China-Japan Friendship Hospital, Yinghua East Road, Chaoyang District, Beijing 100029, China

## Abstract

**Objective:**

To investigate the role of soluble interleukin-2R (sIL-2R) in idiopathic inflammatory myopathies (IIM).

**Methods:**

Serum sIL-2R levels were measured in 74 dermatomyositis (DM), 16 immune-mediated necrotizing myopathy (IMNM), 24 rheumatoid arthritis (RA), 20 systemic lupus erythematosus (SLE), and 20 healthy controls (HCs) by chemiluminescent immunometric assay. Clinical features and laboratory data were collected from electronic medical record. Disease activity was evaluated by using physician global disease activity and myositis disease activity assessment visual analog scale (MYOACT) on admission. 20 DM patients were followed. Serum sIL-2R levels were analyzed and compared with clinical features, laboratory data, and measures of disease activity.

**Results:**

Serum sIL-2R levels were significantly higher in DM patients than in IMNM patients and HCs (648.8 ± 433.1 U/ml vs. 352.3 ± 126.0 U/ml and 648.8 ± 433.1 U/ml vs. 285.8 ± 101.9 U/ml, respectively; all *P* < 0.001), while there was no significant difference between IMNM and HCs. There were also no significant differences of sIL-2R levels in DM, SLE, and RA. Importantly, serum sIL-2R levels were significantly higher in treatment-naïve or active DM patients than those that are not (1100.9 ± 550.4 U/ml vs. 615.6 ± 330.4 U/ml, *P* = 0.006; 808.8 ± 421.6 U/ml vs. 339.8 ± 103.4 U/ml, *P* < 0.001). DM patients with skin ulcers had significantly higher sIL-2R levels than those without (889.3 ± 509.9 U/ml vs. 640.0 ± 368.7 U/ml, *P* = 0.023). Cross-sectional analysis in DM showed that sIL-2R levels positively correlated with CK, ESR, CRP, ferritin, physician VAS, and MYOACT scores (rho = 0.278, rho = 0.474, rho = 0.469, rho = 0.454, *r* = 0.646, and *r* = 0.600, respectively; all *P* < 0.05), negatively correlated with T cell counts and MMT8 scores (*r* = −0.380, *P* = 0.002; rho = −0.394, *P* = 0.001). Follow-up study showed that changes in sIL-2R levels after treatment correlated with changes in physician VAS and MYOACT scores (*r* = 0.823 and *r* = 0.695, respectively; all *P* < 0.01).

**Conclusion:**

Serum sIL-2R levels were elevated in DM but not in IMNM. Serum sIL-2R could act as a disease activity marker and be associated with ulcerative skin lesions in DM.

## 1. Introduction

The idiopathic inflammatory myopathies (IIM) are a group of autoimmune diseases affecting both adults and children. Based on clinical and histopathological features, they can be divided into polymyositis (PM), dermatomyositis (DM), immune-mediated necrotizing myopathy (IMNM), inclusion body myositis (IBM), and overlap myositis [1]. The skin, muscle, and lung are commonly involved organs. Autoantibodies have been identified in over 50% patients. Myositis-specific autoantibodies (MSAs) are useful biomarkers in clinical practice and associated with unique clinical subtypes [2]. Autoimmunity is believed to have a key role in the pathogenesis of myositis. Peripheral T cell lymphopenia is a clinical phenomenon in some IIM patients and correlated with poor prognosis [3, 4]. Immunohistochemical studies on PM/DM muscle biopsies have demonstrated that T lymphocytes often infiltrate muscle fibers [5]. Dysregulated signal pathways were also found in peripheral blood T cells of IIM [6]. This abnormal behavior of T lymphocytes is a characteristic of the pathogenesis of IIM, although the underlying mechanism remains unclear.

Interleukin 2 (IL-2) plays an important role in both effector and regulatory T cell survival. IL-2 acts via the IL-2 receptor (IL-2R), which consists of three different chains: the private *α*-chain (CD25), and *β* (CD122) and *γ* (CD132) chains shared with other cytokine receptors. IL-2R*α* is expressed on T cells rapidly after activation and exerts its function of inducing T cell proliferation in an autocrine and paracrine manner. Soluble IL-2 receptor (sIL-2R) is generated by the proteolytic cleavage of cell-surface receptor [7], and it is considered a serum marker of T cell activation. Soluble IL-2R binds IL-2 with low affinity, and immunosuppressive function of this molecule was proposed. Some studies [8, 9] suggest that sIL-2R is released as a decoy receptor to block IL-2 from binding to effector T cells, while other study found that sIL-2R could protect IL-2 from degradation and inactivation [10].

As a surrogate maker of T cell activation, an increase of serum sIL-2R has been found in autoimmune diseases [11, 12]. In IIM, elevation of serum sIL-2R has also been reported [13–15], but the correlations with clinical characteristics were not described, especially since the differences between MSA subtypes were not explored. Therefore, we aimed to study sIL-2R in sera of IIM patients and to evaluate its association with myositis-related features.

## 2. Patients and Methods

### 2.1. Study Population

74 DM and 16 IMNM patients admitted to the China-Japan Friendship Hospital between April 2018 and May 2019 were enrolled in our study. DM was classified by 2017 ACR/EULAR IIM criteria [16], and IMNM was classified by ENMC IMNM criteria [17]. Patients with other autoimmune diseases, tumors, and infections were excluded. 20 DM patients were followed for 0.8-15 months. Demographic, clinical, and laboratory data were collected from electronic hospital information system. 24 RA and 20 SLE patients admitted during the same period were included as disease controls. 20 healthy people who came to our hospital for routine physical examinations were recruited as healthy controls. The study was approved by the Ethical Review Committee of China-Japan Friendship Hospital.

### 2.2. Assessment of Disease Activity

Evaluation was performed in all IIM patients on the first admission by experienced rheumatologists blind of serum sIL-2R levels. 20 DM patients were also evaluated during subsequent follow-up visit. Myositis disease activity was evaluated by using physician global disease activity recorded on a 10 cm visual analog scale (VAS) [18] and myositis disease activity assessment visual analog scales (MYOACT) [19] established by the International Myositis Assessment and Clinical Studies (IMACS) group. Inactive disease was defined as meeting the following three criteria: CK ≤ 200 U/L, physician VAS = 0, and MYOACT scores = 0. Otherwise, it was considered active.

### 2.3. Blood Test

Blood samples for sIL-2R test were obtained paired with routine blood test after admission during the first and follow-up visit. The sera were stored at -80°C until assayed. Serum sIL-2R levels were determined by using a commercially available chemiluminescent immunometric assay (IMMULITE/IMMULITE 1000 IL2R) according to the manufacturer's instructions; the reference normal range of sIL-2R was 223-710 U/ml. The routine blood test mainly included complete blood count, serum chemistries, ESR, CRP, serum ferritin, and lymphocyte subpopulation study, which were done in the hospital's clinical laboratory by standard automated methods.

### 2.4. Statistical Analysis

Data were analyzed by using IBM SPSS 19.0. Data were expressed as mean ± standard deviation for continuous variables with normal distribution and median (range) for those without. The normality of data was assessed by one-sample Kolmogorov Smirnov test. The unpaired *t*-test and Mann-Whitney test were used for comparisons between groups. Binary logistic regression was adopted to analyze the independent risk factors for sIL-2R elevation. Results from logistic regression were expressed as an odds ratio (OR) with 95% confidence interval (CI). Pearson's (*r*) and Spearman's (rho) correlation analyses were used to test correlations of data with normal and nonnormal distribution, respectively. We considered correlation strength weak when values are between 0 and ±0.3; moderate between ±0.3 and ±0.5; and strong between ±0.5 and ±1.000. *P* values less than 0.05 were considered statistically significant.

## 3. Results

### 3.1. Patient Characteristics

There were 74 DM and 16 IMNM patients included in this study. 24 RA, 20 SLE, and 20 healthy volunteers were included as controls. The demographic and clinical features of the IIM patients are summarized in Table 1. The mean age was 51.0 years old in DM and 49.1 years old in IMNM. The mean age of HC, SLE, and RA was 48.8 years old, 51.7 years old, and 51.5 years old, respectively. There were no significant age differences between IIM patients and the control groups. 71.6% DM, 81.3% IMNM, 70.8% RA, 75.0% SLE, and 70.0% HCs were female. Median disease duration was 10 months in DM and 11 months in IMNM. 78.3% DM and 87.5% IMNM patients were in active disease. 85.1% DM patients were MSA positive, and anti-MDA5 antibody was the most popular. Of the 16 IMNM patients, 11 were anti-SRP or anti-HMGCR positive. The average physician VAS score was 3. The average MYOACT score was 0.099 in DM and 0.039 in IMNM.

### 3.2. Serum sIL-2R Levels Were Increased in DM, but Not in IMNM

**Figure 1:**
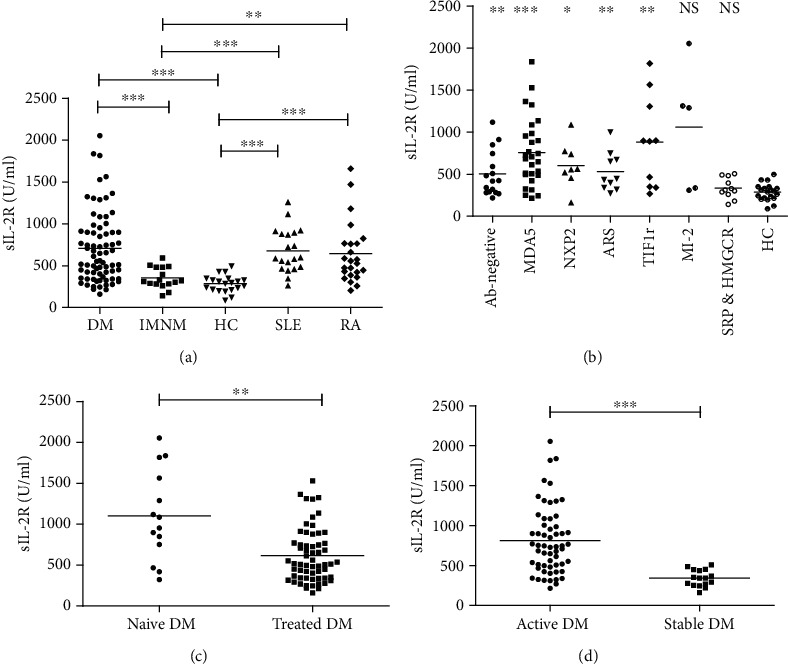
Serum sIL-2R in idiopathic inflammatory myopathies and controls: (a) serum sIL-2R in DM, IMNM, SLE, RA, and HCs; (b) serum sIL-2R in different myositis-specific antibody subgroups compared with HCs; (c) serum sIL-2R in treatment-naïve and previously treated DM patients; (d) serum sIL-2R in active and stable DM patients. DM: dermatomyositis; IMNM: immune-mediated necrotizing myopathy; HC: healthy control; SLE: systemic lupus erythematosus; RA: rheumatoid arthritis. Horizontal bars represent the mean value. ^∗^*P* < 0.05, ^∗∗^*P* < 0.01, and ^∗∗∗^*P* < 0.001; NS: not significant.

Serum levels of sIL-2R were significantly higher in DM patients compared to those of IMNM patients or HCs (648.8 ± 433.1 U/ml vs. 352.3 ± 126.0 U/ml and 648.8 ± 433.1 U/ml vs. 285.8 ± 101.9 U/ml, respectively; all *P* < 0.001), but no significant difference was found between IMNM and HCs (Figure 1(a)). Moreover, serum sIL-2R was significantly higher in DM patients who were treatment-naïve (Figure 1(c)) or in active disease Figure 1(d)) compared to patients with previous treatment or in inactive disease state (1100.9 ± 550.4 U/ml vs. 615.6 ± 330.4 U/ml, *P* = 0.006; 808.8 ± 421.6 U/ml vs. 339.8 ± 103.4 U/ml, *P* < 0.001). However, no significant difference was found between DM, SLE (677.6 ± 261.5 U/ml), and RA (642.0 ± 367.8 U/ml) patients (Figure 1(a)). MSAs are useful in clinical practice and often associated with unique clinical subtypes, so we compared different MSA subgroups with HCs. Serum sIL-2R levels in patients with anti-SRP and anti-HMGCR had no significant difference compared with HCs (334.7 ± 122.4 U/ml vs. 285.8 ± 101.9 U/ml, *P* = 0.243). Serum sIL-2R levels in anti-Mi-2 antibody subgroup tend to be higher than those in HCs (*P* = 0.079), while all the other MSA subgroups had significantly higher sIL-2R levels (Figure 1(b)).

### 3.3. Serum sIL-2R Levels Correlated with Skin Ulcers in DM Patients

Associations between serum sIL-2R levels and clinical manifestations were evaluated in DM patients. We found that serum sIL-2R levels were significantly higher in patients with skin ulcers than those without (889.3 ± 509.9 U/ml vs. 640.0 ± 368.7 U/ml, *P* = 0.023) (Table 2). As skin ulcer could be confounded by disease activity state in DM, the patients were divided into 2 groups with an IL-2R cutoff value of 710 U/ml (the reference upper normal limit) and binary logistic analysis was done. We found that skin ulcer was an independent predictive factor of IL-2R elevation (OR 5.1 (1.3-21.0), *P* = 0.023). As regards muscle weakness, arthritis, dysphagia, interstitial lung disease (ILD), and other skin lesions, no significant differences were observed between these patient subgroups (Table 2).

### 3.4. Serum sIL-2R Levels Correlated with Laboratory Data in DM

**Figure 2:**
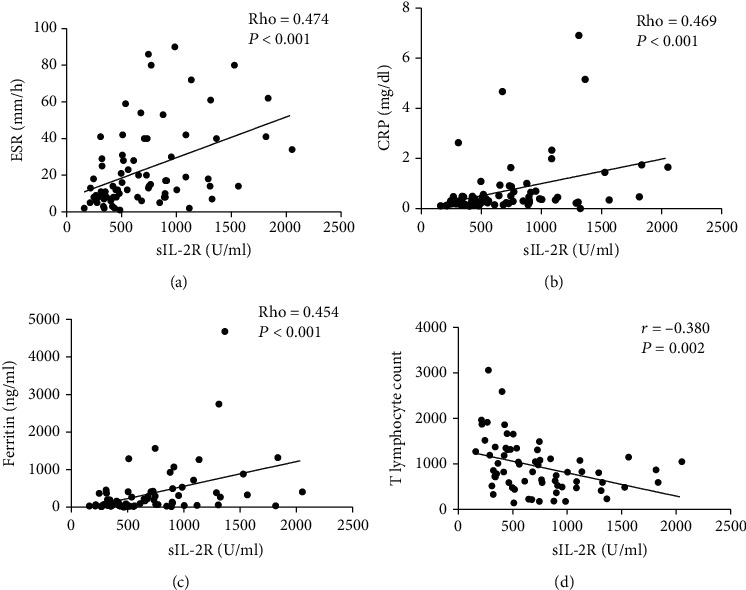
Correlation of serum sIL-2R levels with laboratory data in DM. Correlation of serum sIL-2R levels with (a) erythrocyte sedimentation rate (ESR), (b) C-reactive protein (CRP), (c) serum ferritin, and (d) T lymphocyte counts.

Correlation analysis between serum sIL-2R levels and laboratory data was also conducted. We found that serum sIL-2R levels were moderately positively correlated with erythrocyte sedimentation rate (ESR), C-reactive protein (CRP), and serum ferritin (rho = 0.474, rho = 0.469, and rho = 0.454, respectively; all *P* < 0.001) (Figures 2(a)–2(c)). A weak positive correlation was found between sIL-2R and CK levels (rho = 0.278, *P* = 0.017). Serum sIL-2R is considered to be a marker of T cell activation, so we also analyzed the correlation between sIL-2R levels and peripheral blood lymphocyte subset counts and found a moderate negative correlation between sIL-2R and T lymphocyte counts (*r* = −0.380, *P* = 0.002) (Figure 2(d)).

### 3.5. Changes in Serum sIL-2R Levels and Their Correlations with DM Activity

**Figure 3:**
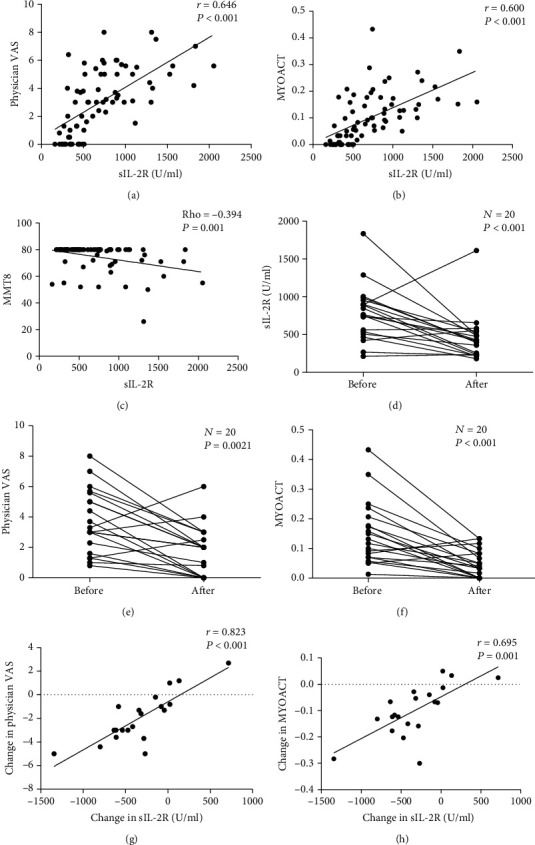
Correlation between serum sIL-2R levels and DM disease activity; (a) correlation between serum sIL-2R levels and physician VAS; (b) correlation between serum sIL-2R levels and MYOACT scores; (c) correlation between serum sIL-2R levels and MMT8; (d) serum sIL-2R levels before and after treatment; (e) MYOACT scores before and after treatment; (f) physician VAS before and after treatment; (g) changes in sIL-2R levels correlated with changes in physician VAS; (h) changes in sIL-2R levels correlated with changes in MYOACT score.

Furthermore, we explored the correlation between serum sIL-2R levels and measures of DM activity. Physician VAS, MYOACT scores, and MMT8 were evaluated at the time of blood sampling. In a cross-sectional study of the 74 DM patients, we found that serum sIL-2R levels were strong positively correlated with physician VAS (*r* = 0.646, *P* < 0.001) and MYOACT scores (*r* = 0.600, *P* < 0.001) (Figures 3(a) and 3(b)). A moderate negative correlation was found between serum sIL-2R levels and MMT8 scores (rho = −0.394, *P* = 0.001) (Figure 3(c)).

20 DM patients were followed for a median of 6 months (range 0.8-15months). Clinical evaluation and serum test were done at each follow-up visit. Serum sIL-2R levels were decreased significantly after immunosuppressive treatment (*P* < 0.001) (Figure 3(d)). MYOACT and physician VAS scores were also decreased (*P* < 0.01) (Figures 3(e) and 3(f)). Changes in serum sIL-2R levels were strongly positively correlated with changes in physician VAS and MYOACT scores (*r* = 0.823 and *r* = 0.695, respectively; all *P* < 0.01) (Figures 3(g) and 3(h)). Notably, 3 followed patients with anti-MDA5 antibody had skin ulcers, the ulcers healed after treatment, and sIL-2R also decreased.

## 4. Discussion

Consistent with previous studies [13, 14, 20], serum sIL-2R levels were increased in DM patients compared to healthy controls. A previous study also found higher sIL-2R levels in PM and reported no difference between DM and PM [13]. Our study involved 16 IMNM patients, 14 of which could be classified as PM by Bohn and Peter criteria, but we found no significant difference between IMNM and HCs. This may partially be explained by the use of different classification criteria. According to Bohn and Peter criteria, the PM patients in the previous study would be of great diversity, including PM, IMNM, and antisynthetase syndrome by the latest criteria. In accordance with other studies [21], the IMNM group had fewer extramuscular features and more severe muscle involvement than DM, so the MYOACT score which represents extramuscular activity was lower in IMNM but the global activity scores are equal between the two groups. The lower sIL-2R level in IMNM may be influenced by the different organ involvement. The lack of sIL-2R elevation in serum is consistent with the absence of significant inflammatory infiltrates in IMNM muscle tissue compared with DM [17]. This result suggests that T cell may play a different role in the pathogenesis of IMNM. But the sample number of IMNM in our study was limited, and more patients are needed to verify the result.

Cutaneous ulcer is characteristic of DM patients with anti-MAD5 antibody and associated with poor prognosis [22, 23]. In clinical analysis, we found that DM patients with cutaneous ulcers had significantly higher sIL-2R levels than those without. A previous study indicated that IL-2 plays a critical role in skin inflammation [24]. Elevated serum sIL-2R levels have been reported in other inflammatory skin disease [25, 26]. Perivascular infiltration of activated T cells in the dermis is a crucial feature in the histological diagnosis of dermatomyositis [27]. As a maker of T cell activation, a linkage between serum sIL-2R and DM skin lesions may be established. Interstitial lung disease is the main complication of DM and predicts poor prognosis. Injection of IL-2 and IL-18 could induce ILD in animal model [28]. Elevation of sIL-2R in bronchoalveolar lavage from patients with systemic sclerosis has been reported, but no difference was found between the patients with or without ILD [29]. We also found no significant difference of serum sIL-2R levels in DM patients with or without ILD.

Our previous study has found that regulatory T cell-related cytokine IL-35 was increased in IIM and could act as a disease activity marker [30]. This promotes us to explore whether sIL-2R could also predict disease activity. We found that sIL-2R varied with disease activity, and significantly increased levels were found during the active phase of DM. Cross-section analysis showed that Serum sIL-2R levels had good correlation with disease activity as represented by MYOACT and physician VAS scores. Serum sIL-2R could be influenced by treatment [31]. Gottfried et al. [32] found elevation of sIL-2R levels in DM patients and reverted to normal after response to IVIG treatment. In our study, we found that treatment-naïve patients had the higher sIL-2R. Follow-up studies also showed decreased serum sIL-2R levels after disease remission, and the changes in sIL-2R levels after treatment correlated with the changes in MYOACT and physician VAS scores. Serum sIL-2R also correlated with other traditional serum makers of disease activity, including CK, ESR, CRP, and serum ferritin. These data suggest that increased serum sIL-2R could act as a disease activity marker in clinical practice.

Serum sIL-2R is a marker of T lymphocyte activation, but the lymphocyte count was significantly negatively correlated to sIL-2R level in our study. One explanation is that sIL-2R may come from the infiltrated inflammatory cells in tissues but not in peripheral blood. Another possibility is increased T lymphocyte death after their activation by IL-2 [33, 34]. Our previous studies [35, 36] reported increased apoptosis in peripheral blood T cells from PM/DM patients. But the exact origin of serum sIL-2R and the reason of lymphopenia in DM still need to be explored.

The limitation of our study is that we did not explore IL-2R expression in peripheral mononuclear cells and muscle or skin tissue, so we did not know the exact source of sIL-2R. Another limitation is that PM patients were not included in our study and IMNM patient number was limited. Further studies are required to explore the sIL-2R difference between PM and IMNM.

In conclusion, our study showed that serum sIL-2R levels were elevated in DM but not in IMNM patients. Serum sIL-2R could be a disease activity marker and associated with ulcerative skin lesion in DM. Further study is needed to explore the source of serum sIL-2R and to clarify its role in the pathogenesis of DM.

## Figures and Tables

**Table 1:** Demographic and clinical characteristics of IIM patients.

Characteristic	DM (*n* = 74)	IMNM (*n* = 16)	*P*
Age (years)	51.0 ± 11.2	49.1 ± 16.8	0.682
Female, no. (%)	53 (71.6%)	13 (81.3%)	0.544
Disease duration, median (range) (months)	10 (1-604)	11 (2-120)	0.804
Active disease state, no. (%)	58 (78.3%)	14 (87.5%)	0.511
Muscle weakness, no. (%)	49 (66.2%)	16 (100%)	0.004^∗∗^
Heliotrope rash, no. (%)	44 (59.5%)	0	<0.001^∗∗∗^
V sign, no. (%)	39 (52.7%)	0	<0.001^∗∗∗^
Gottron's papules/sign, no. (%)	51 (68.9%)	2 (12.5%)	0.001^∗∗^
Mechanic's hands, no. (%)	31 (41.9%)	0	<0.001^∗∗∗^
Ulcers, no. (%)	20 (27.0%)	1 (6.25%)	0.105
Calcinosis, no. (%)	5 (6.8%)	0	0.581
Raynaud's phenomenon, no. (%)	2 (2.7%)	1 (6.25%)	0.474
Arthritis, no. (%)	29 (39.2%)	1 (6.25%)	0.017^∗^
Dysphagia, no. (%)	19 (25.7%)	5 (31.3%)	0.756
Interstitial lung disease, no. (%)	51 (68.9%)	7 (43.8%)	0.083
Myositis-specific autoantibody positive, no. (%)	63 (85.1%)	11 (68.8%)	0.150
Anti-MDA5	28 (37.8%)	0	—
Anti-ARS	10 (13.5%)	0	—
Anti-NXP2	8 (10.8%)	0	—
Anti-SRP	0	8 (50%)	—
Anti-HMGCR	0	3 (18.8%)	—
Anti-Mi-2	5 (6.8%)	0	—
Anti-Tif1*γ*	10 (13.5%)	0	—
Anti-SAE	2 (2.7%)	0	—
Creatine phosphokinase (U/L)	59 (12-18144)	1642 (71-4234)	<0.001^∗∗∗^
MMT8 (0-80)	80 (72-80)	68 (51-80)	0.003^∗∗^
Physician VAS (0-10)	3.0 ± 2.3	3.0 ± 2.2	0.987
MYOACT score (0-1)	0.099 ± 0.093	0.039 ± 0.058	0.038^∗^

^∗^ *P* < 0.05, ^∗∗^*P* < 0.01, and ^∗∗∗^*P* < 0.001.

**Table 2:** Serum sIL-2R levels in DM patients with different clinical manifestations.

Clinical manifestations	With (U/ml)	Without (U/ml)	*P* value
Muscle weakness	700.4 ± 416.8	721.1 ± 442.9	0.844
Skin lesion			
Gottron's papules/sign	753.8 ± 454.6	604.4 ± 327.7	0.161
Heliotrope rash	734.0 ± 406.7	668.4 ± 449.6	0.516
Mechanic's hands	800.7 ± 451.1	640.1 ± 392.8	0.107
Ulcers	889.3 ± 509.9	640.0 ± 368.7	0.023∗
V sign	740.9 ± 494.6	670.0 ± 328.3	0.466
Calcinosis	533.4 ± 178.7	720.0 ± 433.2	0.344
Arthritis	656.6 ± 391.6	740.1 ± 443.0	0.411
Dysphagia	767.9 ± 459.9	686.5 ± 411.6	0.473
Interstitial lung disease	655.7 ± 371.8	822.0 ± 509.1	0.118

^∗^ *P* < 0.05.

## Data Availability

The data used to support the findings of this study are available from the corresponding author upon request.

## References

[1] Selva-O'Callaghan A., Pinal-Fernandez I., Trallero-Araguas E., Milisenda J. C., Grau-Junyent J. M., Mammen A. L. (2018). Classification and management of adult inflammatory myopathies. *The Lancet Neurology*.

[2] Lundberg I. E. (2017). New tools for diagnosis and therapy. *Nature reviews Rheumatology*.

[3] Chen F., Wang D., Shu X., Nakashima R., Wang G. (2012). Anti-MDA5 antibody is associated with A/SIP and decreased T cells in peripheral blood and predicts poor prognosis of ILD in Chinese patients with dermatomyositis. *Rheumatology international*.

[4] Wang D. X., Lu X., Zu N. (2012). Clinical significance of peripheral blood lymphocyte subsets in patients with polymyositis and dermatomyositis. *Clinical rheumatology.*.

[5] Malmstrom V., Venalis P., Albrecht I. (2012). T cells in myositis. *Arthritis research & therapy*.

[6] Shimojima Y., Matsuda M., Ishii W., Kishida D., Sekijima Y. (2017). T-cell receptor-mediated characteristic signaling pathway of peripheral blood T cells in dermatomyositis and polymyositis. *Autoimmunity*.

[7] Rubin L. A., Nelson D. L. (1990). The soluble interleukin-2 receptor: biology, function, and clinical application. *Annals of Internal Medicine*.

[8] Lindqvist C. A., Christiansson L. H., Simonsson B., Enblad G., Olsson-Stromberg U., Loskog A. S. (2010). T regulatory cells control T-cell proliferation partly by the release of soluble CD25 in patients with B-cell malignancies. *Immunology*.

[9] Gooding R., Riches P., Dadian G., Moore J., Gore M. (1995). Increased soluble interleukin-2 receptor concentration in plasma predicts a decreased cellular response to IL-2. *British journal of cancer*.

[10] Kobayashi H., Tagaya Y., Han E. S. (1999). Use of an antibody against the soluble interleukin 2 receptor *α* subunit can modulate the stability and biodistribution of interleukin-2. *Cytokine*.

[11] Eurelings L. E. M., Miedema J. R., Dalm V. A. S. H. (2019). Sensitivity and specificity of serum soluble interleukin-2 receptor for diagnosing sarcoidosis in a population of patients suspected of sarcoidosis. *PLoS One*.

[12] Handa T., Matsui S., Yoshifuji H. (2018). Serum soluble interleukin-2 receptor as a biomarker in immunoglobulin G4-related disease. *Modern Rheumatology*.

[13] Tournadre A., Dubost J. J., Soubrier M. (2014). Soluble IL-2 receptor: a biomarker for assessing myositis activity. *Disease Markers*.

[14] Tokano Y., Kanai Y., Hashimoto H., Okumura K., Hirose S. (1992). Soluble interleukin 2 receptors in patients with polymyositis/dermatomyositis. *Annals of the Rheumatic Diseases*.

[15] Kobayashi I., Ono S., Kawamura N., Okano M., Kobayashi K. (2001). Elevated serum levels of soluble interleukin-2 receptor in juvenile dermatomyositis. *Pediatrics international : official journal of the Japan Pediatric Society.*.

[16] Lundberg I. E., Tjärnlund A., Bottai M. (2017). 2017 European League Against Rheumatism/American College of Rheumatology classification criteria for adult and juvenile idiopathic inflammatory myopathies and their major subgroups. *Annals of the Rheumatic Diseases*.

[17] Allenbach Y., Mammen A. L., Benveniste O. (2018). 224th ENMC International Workshop:: clinico-sero-pathological classification of immune-mediated necrotizing myopathies Zandvoort, The Netherlands, 14-16 October 2016. *Neuromuscular Disorders*.

[18] Rider L. G., Feldman B. M., Perez M. D. (1997). Development of validated disease activity and damage indices for the juvenile idiopathic inflammatory myopathies: I. Physician, parent, and patient global assessments. Juvenile Dermatomyositis Disease Activity Collaborative Study Group. *Arthritis and Rheumatism*.

[19] Isenberg D. A., Allen E., Farewell V. (2004). International consensus outcome measures for patients with idiopathic inflammatory myopathies. Development and initial validation of myositis activity and damage indices in patients with adult onset disease. *Rheumatology*.

[20] Mielnik P., Chwalinska-Sadowska H., Wiesik-Szewczyk E., Maslinski W., Olesinska M. (2012). Serum concentration of interleukin 15, interleukin 2 receptor and TNF receptor in patients with polymyositis and dermatomyositis: correlation to disease activity. *Rheumatology international*.

[21] Pinal-Fernandez I., Casal-Dominguez M., Mammen A. L. (2018). Immune-mediated necrotizing myopathy. *Current Rheumatology Reports*.

[22] Narang N. S., Casciola-Rosen L., Li S., Chung L., Fiorentino D. F. (2015). Cutaneous ulceration in dermatomyositis: association with anti-melanoma differentiation-associated gene 5 antibodies and interstitial lung disease. *Arthritis Care & Research*.

[23] Ishigaki K., Maruyama J., Hagino N. (2013). Skin ulcer is a predictive and prognostic factor of acute or subacute interstitial lung disease in dermatomyositis. *Rheumatology International*.

[24] Ju S. T., Sharma R., Gaskin F., Fu S. M. (2012). IL-2 controls trafficking receptor gene expression and Th2 response for skin and lung inflammation. *Clinical immunology*.

[25] Caixia T., Hongwen F., Xiran L. (1999). Levels of soluble interleukin-2 receptor in the sera and skin tissue fluids of patients with vitiligo. *Journal of Dermatological Science*.

[26] De Rie M. A., Zonneveld I. M., Witkamp L., Van Lier R. A., Out T. A., Bos J. D. (1996). Soluble interleukin-2 receptor (sIL-2R) is a marker of disease activity in psoriasis: a comparison of sIL-2R, sCD27, sCD4, sCD8 and sICAM-1. *Acta Dermato-Venereologica*.

[27] Dourmishev L. A., Wollina U. (2006). Dermatomyositis: immunopathologic study of skin lesions. *Acta dermatovenerologica Alpina, Pannonica, et Adriatica*.

[28] Segawa S., Goto D., Yoshiga Y. (2011). Involvement of NK 1.1-positive *γδ*T cells in interleukin-18 plus interleukin-2-induced interstitial lung disease. *American Journal of Respiratory Cell and Molecular Biology*.

[29] Martinez J. A., Nishimura C., Guatura S. B., Sato E., King T. E. (2001). Elevation of soluble interleukin-2 receptor levels in the bronchoalveolar lavage from patients with systemic sclerosis. *Rheumatology International*.

[30] Yin L., Ge Y., Yang H. (2016). The clinical utility of serum IL-35 in patients with polymyositis and dermatomyositis. *Clinical Rheumatology*.

[31] Nishioka A., Tsunoda S., Abe T. (2018). Serum neopterin as well as ferritin, soluble interleukin-2 receptor, KL-6 and anti-MDA5 antibody titer provide markers of the response to therapy in patients with interstitial lung disease complicating anti-MDA5 antibody-positive dermatomyositis. *Modern Rheumatology*.

[32] Gottfried I., Seeber A., Anegg B., Rieger A., Stingl G., Volc-Platzer B. (2000). High dose intravenous immunoglobulin (IVIG) in dermatomyositis: clinical responses and effect on sIL-2R levels. *European Journal of Dermatology*.

[33] Lenardo M. J. (1991). lnterleukin-2 programs mouse *αβ* T lymphocytes for apoptosis. *Nature*.

[34] Zheng L., Trageser C. L., Willerford D. M., Lenardo M. J. (1998). T cell growth cytokines cause the superinduction of molecules mediating antigen-induced T lymphocyte death. *Journal of immunology*.

[35] Shu X., Chen F., Peng Q. (2017). Potential role of autophagy in T‑cell survival in polymyositis and dermatomyositis. *Molecular Medicine Reports*.

[36] Zhang L., Xia Q., Li W. (2019). The RIG-I pathway is involved in peripheral T cell lymphopenia in patients with dermatomyositis. *Arthritis Research & Therapy*.

